# Biofortification of Carrot (*Daucus carota* L.) with Iodine and Selenium in a Field Experiment

**DOI:** 10.3389/fpls.2016.00730

**Published:** 2016-05-27

**Authors:** Sylwester Smoleń, Łukasz Skoczylas, Iwona Ledwożyw-Smoleń, Roksana Rakoczy, Aneta Kopeć, Ewa Piątkowska, Renata Bieżanowska-Kopeć, Aneta Koronowicz, Joanna Kapusta-Duch

**Affiliations:** ^1^Unit of Plant Nutrition, Institute of Plant Biology and Biotechnology, Faculty of Biotechnology and Horticulture, University of Agriculture in Krakow, KrakówPoland; ^2^Department of Fruit, Vegetable and Mushroom Processing, Faculty of Food Technology, University of Agriculture in Krakow, KrakówPoland; ^3^Unit of Biochemistry, Institute of Plant Biology and Biotechnology, Faculty of Biotechnology and Horticulture, University of Agriculture in Krakow, KrakówPoland; ^4^Department of Human Nutrition, Faculty of Food Technology, University of Agriculture in Krakow, KrakówPoland

**Keywords:** selenomethionine, transfer factor, mineral nutrition, biofortification target, enrichment of plants, beneficial element

## Abstract

The low content of iodine (I) and selenium (Se) forms available to plants in soil is one of the main causes of their insufficient transfer in the soil-plant-consumer system. Their deficiency occurs in food in the majority of human and farm animal populations around the world. Both elements are classified as beneficial elements. However, plant response to simultaneous fertilization with I and Se has not been investigated in depth. The study (conducted in 2012–2014) included soil fertilization of carrot cv. “Kazan F_1_” in the following combinations: (1) Control; (2) KI; (3) KIO_3_; (4) Na_2_SeO_4_; (5) Na_2_SeO_3_; (6) KI+Na_2_SeO_4_; (7) KIO_3_+Na_2_SeO_4_; (8) KI+Na_2_SeO_3_; (9) KIO_3_+Na_2_SeO_3_. I and Se were applied twice: before sowing and as top-dressing in a total dose of 5 kg I⋅ha^-1^ and 1 kg Se⋅ha^-1^. No negative effects of I and Se fertilization were noted with respect to carrot yield. Higher accumulation and the uptake by leaves and storage roots of I and Se were obtained after the application of KI than KIO_3_, as well as of Na_2_SeO_4_ than Na_2_SeO_3_, respectively. Transfer factor values for leaves and roots were about a dozen times higher for Se than for I. Selenomethionine content in carrot was higher after fertilization with Na_2_SeO_4_ than with Na_2_SeO_3_. However, it was the application of Na_2_SeO_3_, KI+Na_2_SeO_3_ and KIO_3_+Na_2_SeO_3_ that resulted in greater evenness within the years and a higher share of Se from selenomethionine in total Se in carrot plants. Consumption of 100 g f.w. of carrots fertilized with KI+Na_2_SeO_3_ and KIO_3_+Na_2_SeO_3_ can supply approximately or slightly exceed 100% of the Recommended Daily Allowance for I and Se. Moreover, the molar ratio of I and Se content in carrot fertilized with KI+Na_2_SeO_3_ and KIO_3_+Na_2_SeO_3_ was the best among the research plots.

## Introduction

For the last few decades selenium (Se) has been considered as a beneficial element for plants – its indispensability for plants has not however been proved. Se can have a growth-promoting effect in many species of plants. Importantly, there is a strict relation between the uptake and metabolism of Se(IV) and P, as well as of Se(VI) and S (Kopsell and Kopsell, 2007; Zhang et al., 2013; Winkel et al., 2015).

The classification of iodine (I) into the group of beneficial elements for plants is not as unanimous as of selenium. There are known reports indicating its positive influence on higher plants (Lehr et al., 1958; Borst-Pauwels, 1961) as well as those underlining the lack of clear understanding and explanation of physiological role of I (Kabata-Pendias, 2011). Differences in iodine classification as a beneficial element result, among others, from the great difficulty in its determination – in our opinion, even greater than in the case of selenium. Another issue is diverse response of individual plant species to iodine application. Smoleń et al. (2016a) presented the hypothesis of genetic diversity with respect to plant reaction to iodine between the old traditional cultivars and those introduced as an effect of ‘green revolution.’ Separate problem are the differences in biomass productivity depending on iodine dose and chemical form: I^-^ and IO_3_^-^ (Blasco et al., 2008, 2012). There are, however, strong indications on positive influence of iodine on nitrogen use efficiency by plants (Blasco et al., 2012; Smoleń and Sady, 2012) or the improvement of tomato fruits (Kiferle et al., 2013; Smoleń et al., 2015).

What needs to be taken into account is the physiological role of I and Se for human and animals. The cause of insufficient transfer of I and Se in the soil-plant-consumer system is the low content of its available forms in the soil. It is also the reason for the limited supply of I and Se in food for several billion people and farm animals worldwide. It is estimated to affect nearly two-thirds of the global human population and is manifested by various diseases and health disorders (Hirschi, 2009; White and Broadley, 2009; Wu et al., 2015).

The relatively cheapest, while still effective way of counteracting the problem could be applying methods of agrotechnical plant biofortification (enrichment) with I and Se, and also with Zn and Fe, whose deficiency is highly widespread round the world as well (Cakmak, 2008; White and Broadley, 2009; Zhao and McGrath, 2009). In perspective, it also seems important to implement biotechnological methods of biofortification for developing new cultivars of plants with more efficient nutrient uptake from soils (White and Broadley, 2009). In comparison to Zn, Fe, and Se (White and Broadley, 2009; Singh et al., 2013; Wu et al., 2015), breeding programs aimed at developing genotypes with increased I content have not found common application (Lyons et al., 2004). Numerous studies, however, have been conducted on agrotechnical methods of plant biofortification/fertilization with individual application of I (Blasco et al., 2010; Kato et al., 2013; Lawson et al., 2015) or Se (Ríos et al., 2008, 2010; Hawrylak-Nowak et al., 2015; Hernández-Castro et al., 2015).

In many countries around the world, I prophylaxis is based on iodization of table salt. Due to the risk of developing various health problems associated with excessive salt consumption, the WHO has recently recommended searching for alternative ways of introducing I into food (WHO, 2004, 2014). In the WHO reports, however, the immense potential of plants with increased I content to support I deficiency prophylaxis was not recognized (WHO, 2014).

The implementation of agrotechnical methods of biofortification for combined fertilization with I and Se is difficult as neither element is a mineral nutrient for plants (Kopsell and Kopsell, 2007; Kabata-Pendias, 2011). In Finland and Malawi, nationwide agrotechnical programs for crop fertilization/biofortification with Se have been conducted (Eurola et al., 2003; Chilimba et al., 2012). On the other hand, enrichment with KIO_3_ of water used for watering fields in Xinjiang Province (China) is an isolated example of soil fertilization with I in agricultural practice (Ren et al., 2008).

In general, irrespective of the cultivation type (field, soilless, hydroponic), the iodide form (I^-^) is more rapidly taken up by roots, and at the same time is more toxic to plants than the iodate form (IO_3_^-^; Blasco et al., 2010; Kato et al., 2013). Some reports, however, have indicated better I absorption from soil after the application of IO_3_^-^ than I^-^ (Smoleń and Sady, 2012; Lawson et al., 2015). In part, this results from the varying preferences of individual plant species with respect to the uptake of I^-^ or IO_3_^-^ ions. Another factor, just as important, is the diverse physical and chemical properties of soils. They determine the sorption, speciation changes of I^-^ and IO_3_^-^ ions or their transformation into volatile I_2_ - I volatilization from soil (Yoshida et al., 1992; Schlegel et al., 2006; Pless et al., 2007). I uptake by roots is highly influenced by Cl concentration in the soil environment. There exists an antagonism between I and Cl, because chloride channels present in the cytoplasmic membrane of plant cells are easily permeable to iodides (Roberts, 2006).

In the case of Se, uptake preferences and plant toxicity of SeO_4_^2-^ and SeO_3_^2-^ also depend on the cultivation type – more precisely, on the environment in which the root system is developed. In the research of Ríos et al. (2008, 2010), the SeO_4_^2-^ form was characterized by better uptake and lower toxicity than SeO_3_^2-^ for lettuce plants cultivated in perlite. In hydroponic cultivation of cucumber the toxicity of the SeO_3_^2-^ form was higher than that of SeO_4_^2-^, despite its lower accumulation in shoots and roots (Hawrylak-Nowak et al., 2015). In natural soils (in field and pot experiments) SeO_4_^2-^ ions are usually more easily taken up and at the same time more toxic to plants than SeO_3_^2-^ ions (Kopsell and Kopsell, 2007; Lavu et al., 2013; Li et al., 2015). This is caused by transformations they undergo in the soil environment, for example, the strong sorption of SeO_3_^2-^ ions with iron hydroxides [Fe(OH)_3_; Elrashidi et al., 1989; Kopsell and Kopsell, 2007]. The mobility and bioavailability of inorganic Se in the environment increase with pH, as well as with decreasing clay and iron oxide content in the soil (Winkel et al., 2015). The possibility of SeO_3_^2-^ and SeO_4_^2-^ uptake by plants from the soil is additionally regulated by plant availability of P and S. In plants, SeO_3_^2-^ ions are transported with the same transport proteins as phosphate, while SeO_4_^2-^ ions are transported with sulfate transporters (Winkel et al., 2015).

The aspects of I and Se interaction due to their simultaneous application have not yet been sufficiently investigated. It is still unknown whether or to what extent increased concentrations of both these elements in the soil environment affect plant growth, development and, finally, yield. Only a few studies in this area have been conducted so far, including hydroponic cultivation of spinach (Zhu et al., 2004) and lettuce (Smoleń et al., 2014). In field conditions, there was only a 1-year study conducted testing soil fertilization with Se+Zn+I in wheat, maize, soybean, potato, canola, and cabbage cultivation (Mao et al., 2014). There are, however, no results from field experiments carried out over more than 1 year documenting the effect of the simultaneous application of various chemical compounds of I and Se on plants.

The research hypothesis was that there is a possibility of increasing I and Se content in carrot plants by conducting simultaneous soil fertilization with mineral forms of these elements. Another one stated that simultaneous application of I and Se has a negative effect on the yield, as even small doses of both elements (not being plant nutrients) can be toxic to plants. It was also assumed that after plant fertilization with Se, a significant increase of organic forms of this element, including selenomethionine, would occur.

The research objective was to determine the effects of soil fertilization with different chemical forms of I (I^-^ and IO_3_^-^) and Se (SeO_3_^2-^ and SeO_4_^2-^) on crop yield, the efficiency of biofortification with these elements and selected chemical properties of carrot plants (*Daucus carota* L.).

## Materials and Methods

### Plant Material and Treatments

In the years 2012–2014, a field study with carrot (*Daucus carota* L.) cv. “Kazan F_1_” cultivation was conducted in Marszowice near Kraków, Poland. Each year, carrot was cultivated on the same farm – on a different site within a single soil complex.

**Table 1:** Selected chemical properties of the 0–30 cm soil layer prior to the experiment in 2012–2014 (*n* = 4).

Parameter	2012	2013	2014
pH_H2O_	6.30	7.77	6.10
EC (dS m^-1^)	0.13	0.12	0.04
Eh (mV)	+220.0	+257.9	+233.5
Iodine (mgkg^-1^)	0.25	0.24	0.25
Selenium (mgkg^-1^)	0.59	0.57	0.60
Organic matter (%)	2.11	2.48	2.25
Particle size fraction (%): sand/silt/loam	4/47/49	2/48/50	4/47/49
Soil texture class	Silty clay (heavy soil)	Silty clay (heavy soil)	Silty clay (heavy soil)

Carrot was cultivated on a heavy soil with a silty clay texture (**Table 1**). In 2012 and 2014 carrot was cultivated on the same field (with a total area of ~2 ha) but on its different parts characterized by various crop rotations within an area of 1 ha (coordinates: 50.1911481 N, 20.0902866 E, 306 masl). Each year, the preceding crop was wheat. Importantly, on the soil site on which carrot was grown in 2014, tobacco was cultivated in 2012, not carrot.

The value of soil pH was higher in 2013, as in that year carrot was cultivated on another field located about 500 m from the field used in 2012 and 2014 (coordinates: 50.1868649 N, 20.0925089 E, 306 masl). Despite the pH differences, soils from the fields under study were characterized by similar I and Se content in each year.

The study included soil fertilization with I and Se in the following combinations: (1) Control; (2) KI; (3) KIO_3_; (4) Na_2_SeO_4_; (5) Na_2_SeO_3_; (6) KI+Na_2_SeO_4_; (7) KIO_3_+Na_2_SeO_4_; (8) KI+Na_2_SeO_3_; (9) KIO_3_+Na_2_SeO_3_. I and Se were applied twice: before sowing (before ridge formation) and as a top-dressing – at canopy closure, each in a dose of 2.5 kg I⋅ha^-1^+ 0.5 kg Se⋅ha^-1^. The total amount of introduced I and Se was 5 kg I⋅ha^-1^ and 1 kg Se⋅ha^-1^, respectively. Pre-sowing fertilization with I and Se was conducted on April 12, 2012, April 18, 2013, and April 04, 2014, and top-dressing application on June 29, 2012, July 3, 2013 and July 4, 2014. I was applied as KI and KIO_3_ (puriss. p.a., Avantor Performance Materials, Gliwice, Poland), and Se as Na_2_SeO_4_ and Na_2_SeO_3_ (puriss. p.a., Sigma–Aldrich Co. LLC, St. Louis, MO, USA). The experiment was arranged in a split-plot design. Each treatment was randomized in four repetitions on 4 m × 6 m (24 m^2^) plots. The total area of the experiment was 864 m^2^.

One day prior to bed formation, based on the results of soil chemical analysis, pre-sowing fertilization with N, P, and K was conducted (along with I and Se application), in order to supplement the soil to the level optimal for carrot: N-100, P-80, and K-200 (in mgdm^-3^ of soil). N fertilization was applied as urea (Zakłady Azotowe “Puławy,” Puławy, Poland), P and N as ammonium phosphate (Grupa Azoty SA, Zakłady Chemiczne “Police,” Police, Poland) and K as 60% potassium salt (Zakład Obrotu Towarami Sp. z o. o., Dwikozy, Poland). Doses of these fertilizers in individual years, respectively, for 2012, 2013, and 2014, were as follows: urea 0.25, 0.10, and 0.25 t⋅ha^-1^, ammonium phosphate 0.80, 1.25, and 1.00 t⋅ha^-1^ as well as potassium salt 0.90, 1.10, and 1.00 t⋅ha^-1^.

Carrots were cultivated in one row on 40 cm wide and 30 cm high raised beds at a seeding rate of 37 seeds⋅m^-1^ (approximately 600,000 seeds per hectare). The seeds were sown on April 19, 2012, April 25, 2013, and April 05, 2014. The carrot roots were harvested on September 26, 2012, September 11, 2013, and September 9, 2014. During harvest, yield of carrot leaves and storage roots as well as plant density per hectare were determined. Marketable yield consisted of storage roots of cylindrical or close-to-cylindrical shape with a head diameter of ≥3 cm, undamaged by pests, not infected by fungi or bacteria, with no fractures and heads greened to a maximum of 0.5 cm. The length of a storage root was 15 cm minimum. At harvest, approximately 10 kg samples of carrot storage roots were chosen from each of the four plots (replications) for laboratory analysis. Only roots qualifying as marketable yield were taken for further chemical analysis.

During carrot harvest, soil samples from the layers 0–30 cm, 30–60 cm, and 60–90 cm were also collected separately for each of the research plots. Soil samples from each layer from each of the four plots (replications) were collected by soil drill (diameter 3 cm) for laboratory analysis. Additionally, before cultivation, eight individual samples from the whole area of the experimental fields were randomly collected (**Table 1**).

### Plant Analysis

Samples of carrot leaves and storage roots were dried at 70°C in a laboratory dryer with forced air circulation and ground in a Pulverisette 14 Fritsch (Idar-Oberstein, Germany) variable speed rotor mill, using a 0.5 mm sieve. Samples thus prepared were subsequently analyzed with respect to the content of I and Se with an ICP-OES spectrometer (Prodigy, Leeman Labs, New Hampshire, MA, USA) and selenomethionine (SeMet) using a capillary electrophoresis (CE) analyzer PA 800 Plus CE system with DAD detection (Beckman Coulter, Indianapolis, IN, USA)

Iodine content in leaf and root samples was determined with the application of the cold vapor I_2_ generation (CVG) technique (Vtorushina et al., 2008, 2009). Air-dried plant samples (0.5 g) were digested in a mixture of super-pure 10 cm^3^ 65% HNO_3_ (Merck, Whitehouse, Station, NJ, USA) and 0.8 cm^3^ 70% HClO_4_ (Avantor Performance Materials, Gliwice, Poland) in a CEM MARS-5 Xpress (CEM World Headquarters, Matthews, NC, USA) microwave system using Teflon vessels. The process consisted of four steps with a gradual increase of temperature: 60, 80, and 100°C (each step involved 10 min of warming plus 5 min maintenance at the set temperature) and 130°C (10 min + 15 min). After digestion, solutions were transferred into a volume of 25 cm^3^ with redistilled water. Measurements using the CVG technique required the application of a gas/liquid separator using 30% hydrogen peroxide (Avantor Performance Materials, Gliwice, Poland) for isolating volatile I_2_ during measurement with an ICP-OES spectrometer (Vtorushina et al., 2008).

Selenium content was analyzed after sample digestion in nitric acid (Pałsawski and Migaszewski, 2006). Samples of air-dried carrot leaves and storage roots (0.5 g) were digested at 200°C (15 min of warming plus 15 min maintenance at the set temperature) in 10 cm^3^ 65% super-pure HNO_3_ (Merck, Whitehouse, Station, NJ, USA) using a CEM MARS-5 Xpress microwave system. Samples were then transferred to the final volume of 25 cm^3^ using double-distilled water and analyzed with an ICP-OES spectrometer.

The SeMet content in leaf and root samples was determined using the following procedure. In 10 cm^3^ Falcon tubes, 5 cm^3^ of solution containing 40 mg protease and 20 mg lipase in demineralized water were added to 0.25 g of air-dried plant samples. The samples were incubated for 16 h at 20°C and centrifuged for 15 min at 4500 rpm (Zhao et al., 2011). The supernatants were filtered through 0.25 μm cellulose acetate membrane filters and analyzed using a CE analyzer with DAD detection, at 254 nm. A silica capillary tube with an i.d. of 75 μm, o.d. of 365 μm and a total length of 50 cm was used for the measurements. A negative power supply of -16 kV was applied. The running buffer solution was prepared as proposed by Zhao et al. (2011), containing 30 mmol NaH_2_PO_4_ (Avantor Performance Materials, Gliwice, Poland), 15 mmol Na_2_B_4_O_7_ (Sigma–Aldrich Co. LLC, St. Louis, MO, USA, puriss. p.a.) and 0.2 mmol CTAB (pH 8.80; Sigma–Aldrich Co. LLC, St. Louis, MO, USA, puriss. p.a.). Standards of L(+)-SeMet (Acros Organics, Geel, Belgium) were used for CE calibration.

### Soil Analysis

Before carrot cultivation, soil samples were taken from the 0–30 cm layer, in order to characterize the physical and chemical properties of the soil site (**Table 1**). The results obtained are presented in Section “Plant Material and Treatments.” Prior to carrot cultivation, soil texture was analyzed using the Casagrande method modified by Pruszyński (Komornicki et al., 1991) as well as pH, total soil salinity (EC), redox potential (Eh), and the organic matter content. In soil samples mixed with water (1:2 v/v, soil:H_2_O), pH and Eh were measured potentiometrically and EC was analyzed using a conductivity meter. The organic matter in the soil was determined by the Tiurin method (Nowosielski, 1988; Komornicki et al., 1991).

Iodine and Se content in the soil before and after carrot cultivation was analyzed using the following procedure. Soil samples were dried at 70°C in a laboratory dryer with forced air circulation, ground in a mortar and sieved through a 1 mm sieve. Soil samples (2.5 g) were put into 30 cm^3^ Falcon tubes; 10 cm^3^ of double-distilled water and 1 cm^3^ of 25% TMAH (tetramethylammonium hydroxide - Sigma–Aldrich Co. LLC, St. Louis, MO, USA) were added. After mixing, the samples were incubated for 3 h at 90°C. After incubation, the samples were cooled to a temperature of approximately 20°C and filled to 30 cm^3^ with double-distilled water. After mixing, the samples were centrifuged for 15 min at 4500 rpm. The measurements, using an ICP-OES spectrometer (Prodigy, Leeman Labs, New Hampshire, MA, USA), were conducted in the supernatant (without decanting it). The method described above is our own modified procedure (Smoleń et al., 2016a) for the determination of total I (Yamada et al., 1996) and I and Se (McNally, 2011) content in soil using TMAH. The modification included the application of higher temperature (90°C, not 70°C) and omitting sample filtration after centrifugation. Filtration of samples leads to losses of analyzed elements. The temperature of 90°C is recommended for I determination after sample incubation with TMAH according to PN-EN 15111 (2008).

### ICP-OES Spectrometer Settings for Iodine and Selenium Determination

Analysis of the I and Se content in leaves and storage roots of carrot and soil was conducted using a ICP-OES Prodigy spectrometer (Prodigy, Leeman Labs, New Hampshire, MA, USA). Calibration of the instrument was performed by maintaining the same matrix as for the analyzed samples. For I determination in plant samples using the CVG technique, the most sensitive line of I: I-178.276 nm (with the detection limit of 0.5 μg I⋅dm^-3^) was chosen, as for this method no interference from P affects it (Vtorushina et al., 2009). After alkaline extraction of soil samples (using TMAH), the I-183.038 nm (with the detection limit for solution nebulization of app. 50 μg I⋅dm^-3^) line was used. For soil sample extraction with TMAH I-178.276 nm and I-206.163 spectral lines cannot be used due to the interferences from, respectively: P as well as Zn and Cr. The Se content in both the carrot (leaves and storage roots) and soil samples was analyzed using the Se-169.090 nm line.

### Meteorological Data

**FIGURE 1:**
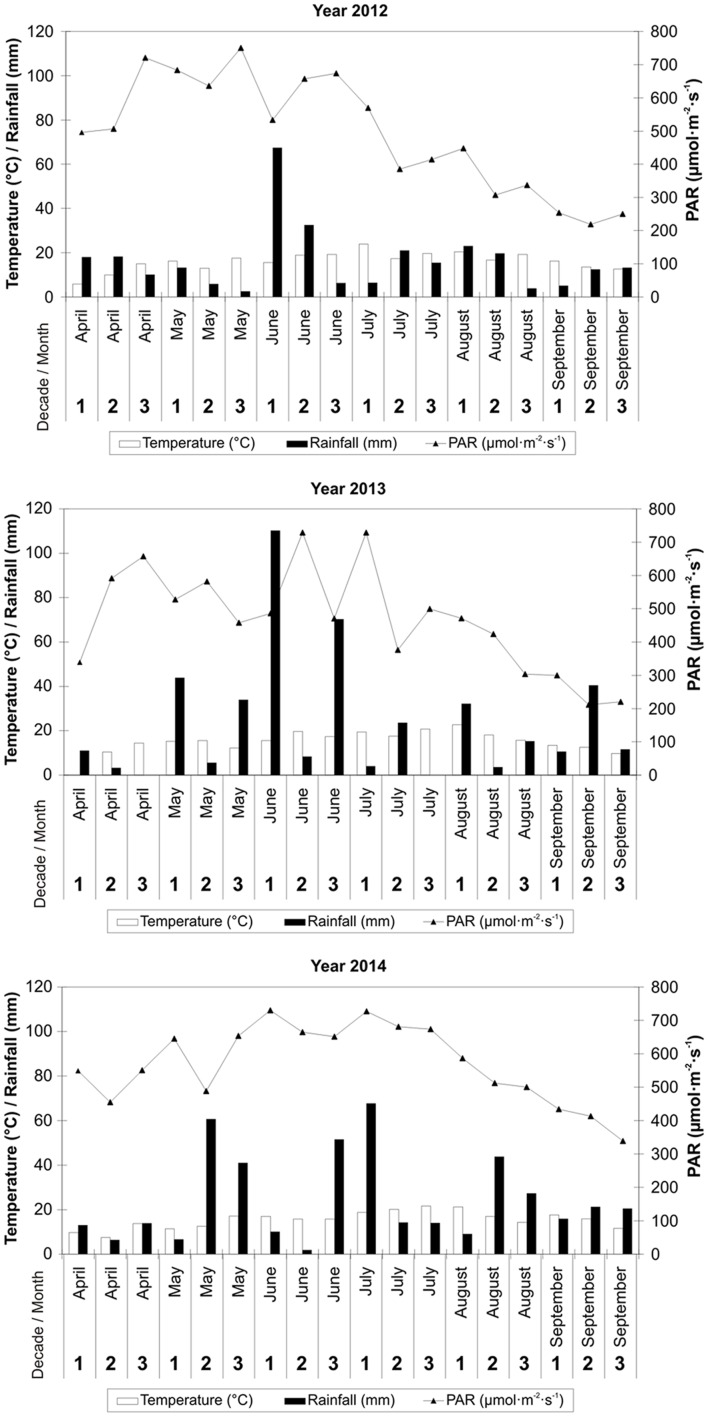
**Meteorological data for carrot cultivation in 2012, 2013, and 2014**.

Each year, carrot was grown from April to September. Meteorological data from dekadal (10 days) periods are presented including the mean daily air temperature, mean daily PAR value and total precipitation (**Figure 1** – data from a HOBO Weather Station). From April to September, the total amount of rainfall was 293.4, 428.5, and 437.9 mm, whereas the mean daily air temperature was 16.1, 15.2, and 15.5°C in 2012, 2013, and 2014, respectively (**Figure 1** – data from a HOBO Weather Station). The average daily PAR value in the period from carrot planting in field to the harvest was: 491.0, 465.3, and 569.6 μmo⋅lm^-2^⋅s^-1^ in 2012, 2013, and 2014, respectively.

For comparison, the mean air temperature for the period 1971–2000 from April to September was 14.3°C, and total precipitation was 435 mm (GUS, 2005). More precisely, in the years 1971–2000, the average monthly air temperature was 8.0°C in April, 13.4°C in May, 16.2°C in June, 17.8°C in July, 17.5°C in August, and 13.2°C in September. In the same period, the average monthly rainfall in the area under study was 50 mm in April, 74 mm in May, 94 mm in June, 81 mm in July, 76 mm in August, and 60 mm in September (GUS, 2005).

The most unfavorable weather conditions during carrot cultivation occurred in 2012 and mainly concerned the amount and distribution of rainfall (**Figure 1**). The total amount of rainfall from April to September 2012 was 141.6 mm lower, while in the years 2013–2014 it oscillated around the mean for the period of 1971–2000. A higher mean daily air temperature was also recorded from April to September 2012 when compared to the same period in 2013 and 2014 - it was 1.8°C higher than the mean from the years 1971–2000. That was caused by heat waves occurring in the summer months of 2012.

With respect to PAR, its highest total was noted in 2014, and at the same time it was the most evenly distributed throughout the entire period of carrot cultivation within all 3 years of the study (**Figure 1**). In the years 2012–2013, large fluctuations in PAR values in the subsequent dekadal periods from April to mid-August were recorded.

### Data Analysis

The I and Se transfer factor (TF) in the soil-plant (carrot leaves or storage roots) system was calculated using the following formula:

$$TF=\frac{C_{plant\,\;dry\,\;weight}}{C_{soil\,\;dry\,\;weight\,\;(concentration\,\;in\,\;the\,\;soil\,\;before\,\;\,\;the\,\;\exp eriment\,+\;fertilization\,)}},$$

with C standing for I and Se content in plant/soil dry matter. Based on the results of crop yield measurement, I and Se determination (and dry weight) in carrot leaves and storage roots, values of I and Se uptake by leaves, storage roots and whole plants of carrot were calculated. Using the results of Se and SeMet content, the percentage ratio of Se present in SeMet in relation to the total Se content in the leaves and storage roots of carrot was calculated.

#### Biofortification Target

The percentage of Recommended Daily Allowance for I (RDA-I) and Se (RDA-Se) supplied from one serving of a 100 g portion of fresh carrot storage roots was calculated. In these calculations, results of I and Se determination in carrot as well as the recommended daily intake of these two elements: 150 μg I and 55 μg Se per day for adults were used (Food and Nutrition Board – Institute of Medicine, 2000; Andersson et al., 2007).

All data were subjected to analysis of variance using the ANOVA module of Statistica 10.0 PL. For determining the significance between the means, the Tukey test was used. The significance was declared at *P* < 0.05.

## Results

### Carrot Yield

**FIGURE 2:**
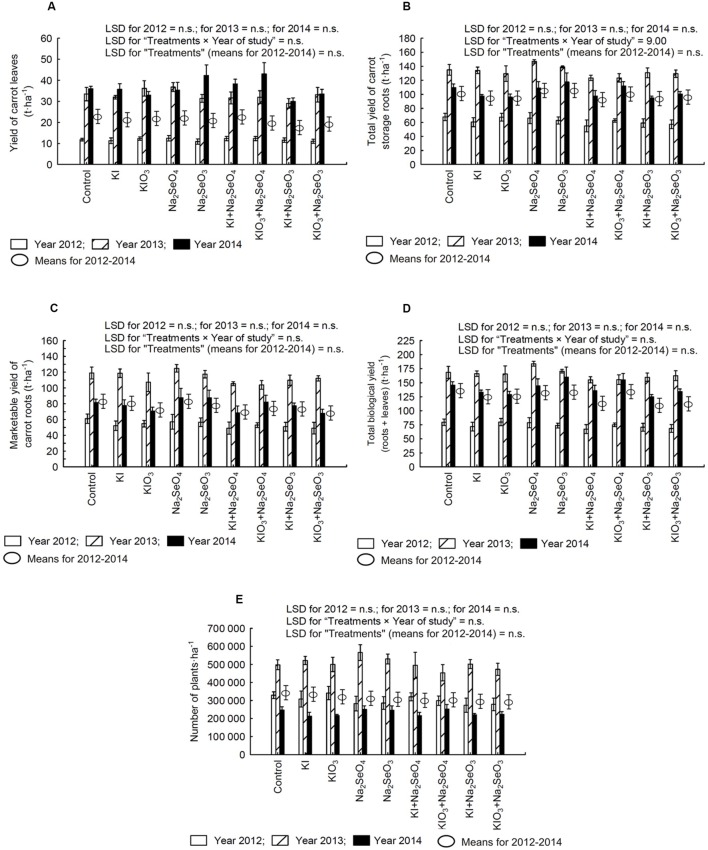
**Effect of iodine and selenium fertilization on yield: of leaves (A), total **(B)**, and marketable yield **(C)** of storage roots as well as the biological yield of carrot (leaves and storage roots) **(D)**, and the number of carrot plants per hectare **(E)** in the years 2012–2014.** LSD, least significant difference; n.s., not significant, *p* < 0.05. Bars indicate standard error; (*n* = 4).

In the following years of the research, no statistically significant effect of I and Se fertilization was observed with respect to all measured yield parameters (**Figures 2A–E**). With the exception of the total yield of carrot storage roots, no significant interaction of treatments × year of study on tested yield parameters was demonstrated (**Figure 2B**). It was mostly affected by substantial differences in total yield of carrot storage roots in the subsequent years. In comparison to the control, the total yield of storage roots in 2012 decreased after the application of Na_2_SeO_4_ and Na_2_SeO_3_, while in other years remained at a level comparable to the control. Only in 2013, simultaneous soil application of I and Se compounds tended to lower the total yield of storage roots as compared to the control

In 2012 and 2014, a lower planting density was observed than in 2013 (**Figure 2E**). However, only in 2012 the carrot leaves and root yield differed from the yield obtained in the years 2013–2014 (**Figures 2A–D**).

### Iodine Content in Carrot Plants

**FIGURE 3:**
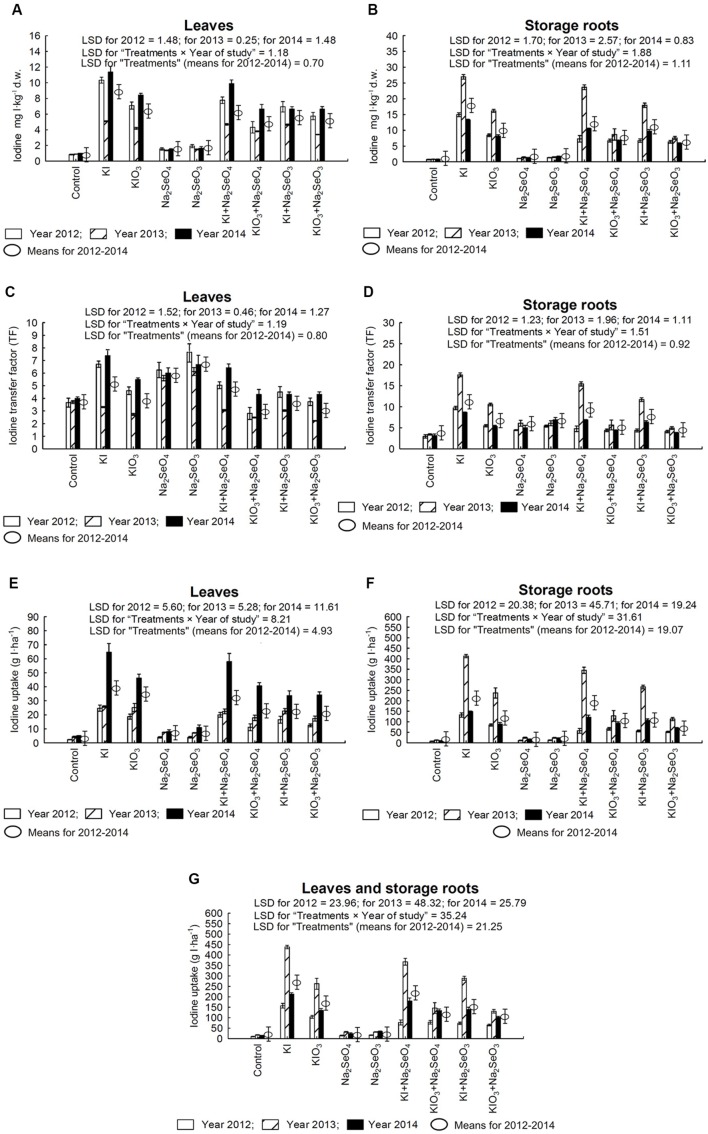
**Iodine concentration in leaves **(A)** and storage roots **(B)**, transfer factor (TF) of iodine to leaves **(C)** and storage roots **(D)** as well as iodine uptake by leaves **(E)**, storage roots **(F)** and whole plants of carrot **(G)** in the years 2012–2014, depending on varying fertilization with iodine and selenium.** LSD, least significant difference, *p* < 0.05. Bars indicate standard error; (*n* = 4).

Fertilization with I and Se had a significant effect on the accumulation of I in carrot leaves and storage roots (**Figures 3A,B**), values of I TF for roots and leaves (**Figures 3C,D**) and I uptake (g I⋅ha^-1^) by leaves, storage roots and whole carrot plants (**Figures 3E–G**).

Each year, the highest I content in carrot leaves and storage roots along with the highest I uptake was characterized by plants fertilized solely with KI (**Figures 3A,B,E–G**). In each year of the research, in combinations with KIO_3_ applied separately and together with Se, plants were fortified with I less effectively than when using KI (**Figures 3A,B**). This observation was further confirmed by lower I uptake by leaves and storage roots when using KIO_3_ rather than KI (**Figures 3E,F**). Simultaneous fertilization with KI/KIO_3_ and Se reduced I content and uptake by leaves and storage roots, in comparison to plants fertilized exclusively with I. To a greater extent, it applied to using the SeO_3_^2-^ form of Se.

Fertilization only with Se in the form of Na_2_SeO_4_ and Na_2_SeO_3_, as compared to the control, caused a substantial increase of I content in leaves and storage roots (**Figures 3A,B**) - however, much lower than after the application of KI and KIO_3_ separately and or with Se. After fertilization with Na_2_SeO_4_ and Na_2_SeO_3_, TF values for I uptake by leaves were at a level similar to or higher than after the application of KI and KIO_3_ separately and in combination with Se (**Figure 3C**). In storage roots, TF values for I in plants from Na_2_SeO_4_ and Na_2_SeO_3_ plots were lower than in plants treated with KI application (**Figure 3D**).

The highest accumulation, TF and I uptake by roots (**Figures 3B,D,F**), and the lowest content and TF of I by carrot leaves were noted in 2013, as compared to 2012 and 2014 (**Figures 3A,C**).

### Selenium and Selenomethionine Content in Carrot Plants

**FIGURE 4:**
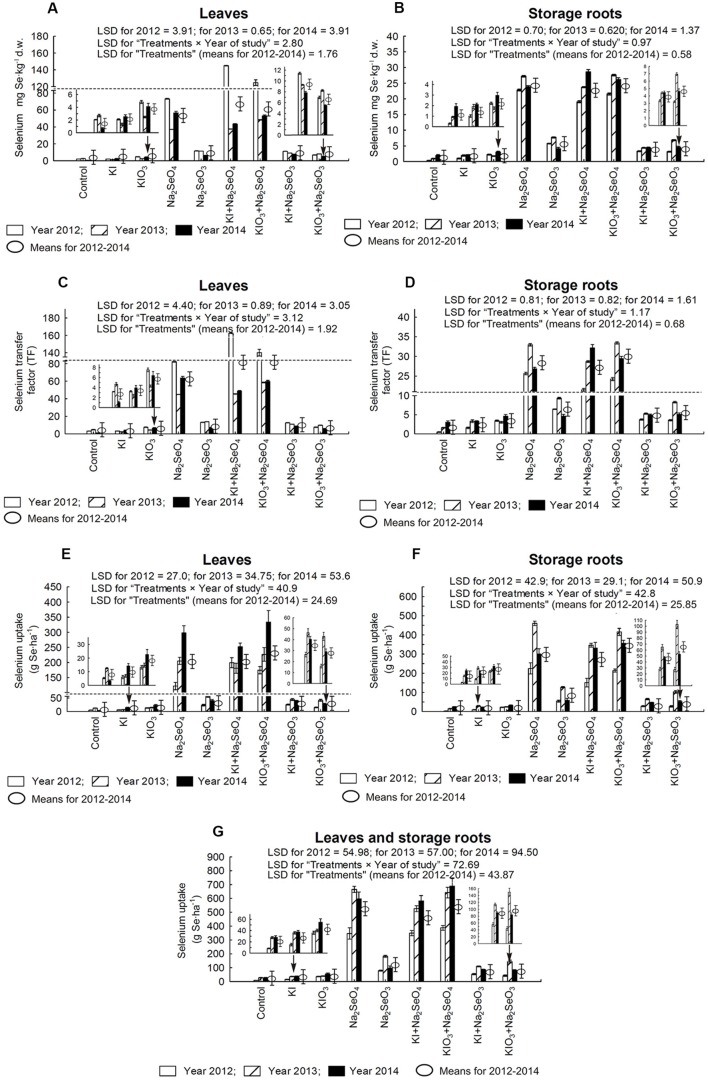
**Selenium concentration in leaves **(A)** and storage roots **(B)**, transfer factor (TF) of selenium to leaves **(C)** and storage roots **(D)** as well as selenium uptake by leaves **(E)**, storage roots **(F)** and whole plants of carrot **(G)** in the years 2012–2014, depending on varying fertilization with iodine and selenium.** LSD, least significant difference, *p* < 0.05. Bars indicate standard error; (*n* = 4).

**FIGURE 5:**
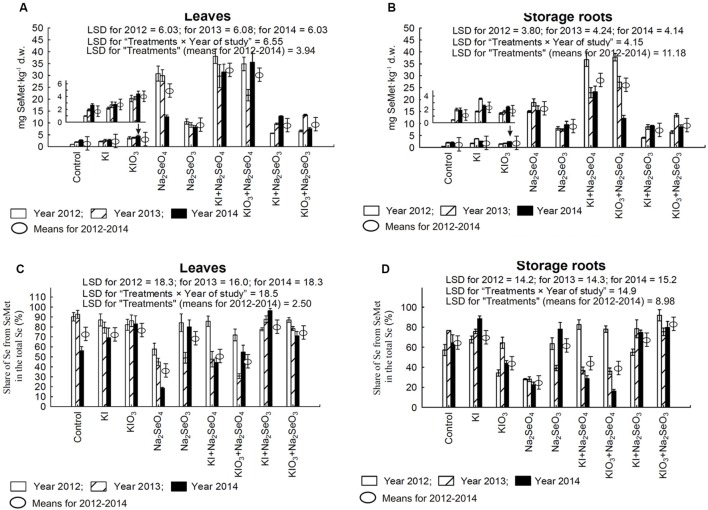
**Selenomethionine (SeMet) concentration in leaves **(A)** and storage roots **(B)** as well as the percentage of total selenium present in SeMet for carrot leaves **(C)** and storage roots **(D)** in the years 2012–2014, depending on varying fertilization with iodine and selenium.** LSD, least significant difference, *p* < 0.05. Bars indicate standard error; (*n* = 4).

In comparison to the control and plots with the application of only I, in each year of the research, a significant increase of Se accumulation, TF values for Se, Se uptake and SeMet content in carrot leaves and storage roots were noted in plants from the plots with Se and Se+I (**Figures 4A–G** and **5A,B**). In plants fertilized with Na_2_SeO_4_ separately or with KI/KIO_3_, the values of these parameters were up to several dozen times higher than in plants fertilized with Na_2_SeO_3_ in the respective combinations.

In relation to the control, fertilization with KI or KIO_3_ alone caused a considerable increase of Se content in storage roots – with the exception of KI application in 2014 (**Figure 4B**). In the case of leaves, a similar effect of soil fertilization with KI and KIO_3_ was noted only in 2014 (**Figure 4A**).

For each year of the research, diverse effects of relations between the application of Na_2_SeO_4_ alone or together with KI and KIO_3_ were noted with respect to Se content, Se TF values, Se uptake and SeMet content in carrot leaves and storage roots (**Figures 4A–G** and **5A,B**). Conversely, fertilization with KI+Na_2_SeO_3_ and KIO_3_+Na_2_SeO_3_, compared to the application of Na_2_SeO_3_ alone, considerably lowered Se content, TF values and uptake - to a greater extent after the application of the I^-^ form of I (**Figures 4A–G**). With respect to SeMet content, no significant interaction of KI and KIO_3_ with Na_2_SeO_3_ in relation to fertilization with Na_2_SeO_3_ alone was, however, observed (**Figures 5A,B**).

The percentage of total Se found in SeMet calculated for carrot leaves and storage roots (further expressed as “Se-SeMet in total Se”) was the parameter with the highest variability within the years (**Figures 5C,D**). Its values for leaves were significantly higher than for roots and varied from 18.4% (combination with Na_2_SeO_4_ in 2014) to 96.3% (KI+Na_2_SeO_3_ in 2014). In storage roots, selenium from selenomethionine accounted for between 16.2% (KIO_3_+Na_2_SeO_4_ in 2014) and 91.8% (KIO_3_+Na_2_SeO_3_ in 2014) of total Se. During the entire 3-year research period a substantial decrease in Se-SeMet in total Se for both leaves and roots was noted after fertilization with Na_2_SeO_4_ (and KI+Na_2_SeO_4_ and KIO_3_+Na_2_SeO_4_ in 2013 and 2014 – **Figures 5C,D**).

### Percentage of Recommended Daily Allowance of Iodine (RDA-I) and Selenium (RDA-Se)

**FIGURE 6:**
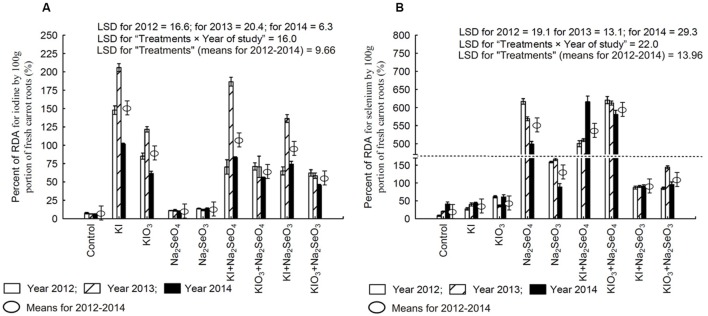
**Percentage of Recommended Daily Allowance (RDA) for iodine **(A)** and selenium **(B)** in 100 g portion of fresh carrot roots in the years 2012–2014, depending on varying fertilization with iodine and selenium.** The dotted line represents a break (shortening) of the scale on the *Y*-axis between 170 and 470. LSD, least significant difference, *p* < 0.05. Bars indicate standard error; (*n* = 4).

Soil fertilization with I and Se compounds substantially increased the percentage of the RDA-I and RDA-Se supplied by the intake of 100 g of fresh carrot roots (**Figures 6A,B**). The RDA-I and RDA-Se values for plants in each plot, and between them, were a highly varied characteristic throughout the research years.

In the case of I, a single portion of 100 g f.w. of carrot fertilized with KI in each research year (as well as KI+Na_2_SeO_4_ and KI+Na_2_SeO_4_ in 2013) would supply more I than recommended for adults (**Figure 6A**). In 2013, due to the application of KIO_3_ alone, a greater than 100% supply of RDA-I by a single portion of carrot was noted. It is significant that I content in a single portion of carrot fertilized with KIO_3_ together with Se (irrespective of its chemical form) every year would supply less than 100% of RDA-I.

In the case of Se, each year a single portion of 100 g f.w. of carrot fertilized with Na_2_SeO_4_, KI+Na_2_SeO_4_ and KIO_3_+Na_2_SeO_4_ would supply Se in a supraoptimal quantity - from 500 to 650% RDA-Se for adults (**Figure 6B**). The use of Na_2_SeO_3_ for biofortification purposes (applied separately and combined with I) affected RDA-Se to a much smaller extent than Na_2_SeO_4_ but its values still oscillated around or exceeded 100%.

It should be underlined that soil fertilization solely with KI or KIO_3_ also caused a significant increase of about 12% of daily consumer allowance of Se supplied with a single portion of fresh carrots when compared to the control – the relation was noted in 2012 and 2013 (**Figure 6B**).

### Iodine and Selenium Content in Soil after Carrot Cultivation

**FIGURE 7:**
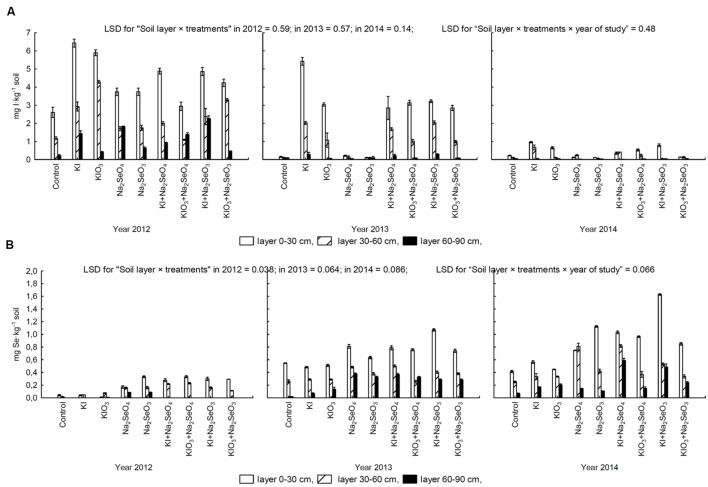
**Concentration of iodine **(A)** and selenium **(B)** in 0–30 cm, 30–60 cm, and 60–90 cm soil layers after carrot cultivation in the years 2012–2014, depending on varying fertilization with iodine and selenium compounds.** LSD, least significant difference, *p* < 0.05. Bars indicate standard error; (*n* = 4).

In the years 2012–2014, a highly varied effect of I and Se fertilization on the content of these two elements in soil after carrot cultivation was noted (**Figures 7A,B**). The highest I and the lowest Se concentrations in soil (total in all layers) were determined in 2012, whereas in 2014 the opposite relations were observed.

In each research year, I content in soil from plots fertilized with this element exceeded that in the control and combinations treated with Se alone (**Figure 7A**). It was highest in the 0–30 cm layer and lowest in the 60–90 cm layer. Similarly in the case of Se, soil fertilized with this element accumulated more Se than from the control combination and those fertilized with KI and KIO_3_ (**Figure 7B**). The content of Se in each layer was in the following order: 0–30 cm > 30–60 cm > 60–90 cm (**Figure 7B**).

## Discussion

### Crop Yield and Iodine and Selenium Uptake by Carrot

In the study conducted by Smoleń et al. (2016b) with lettuce cultivation according to the same experimental design, substantial symptoms of Na_2_SeO_4_ toxicity followed by lower lettuce yield was noted irrespective of additional application of iodine. Therefore it may be concluded that carrot is a species less sensitive, than lettuce, to the SeO_4_^2-^ form of Se. The cause of such an observation may be the lower Se accumulation in carrot plants (two to three times lower than in lettuce leaves – Smoleń et al., 2016b).

The lack of negative response of carrot plants (in terms of yield) to I fertilization may be the consequence of its tolerance to iodine. In a pot experiment carried out in a greenhouse, Dai et al. (2004) did not observe symptoms of toxicity nor reduction of carrot biomass in reaction to increasing doses of KIO_3_ fertilization (0, 1, and 5 mg I⋅kg^-1^ of soil) as opposed to spinach, pak choi and celery. The biochemical and physiological mechanisms responsible for such diverse plant responses to I are not, however, known.

In the present study, iodine uptake by carrot plants was substantially improved when this element was applied as I^-^ than IO_3_^-^. Similar observations were noted for lettuce cultivated in perlite (Blasco et al., 2008). When comparing the absolute values of I TF for carrot with results from other studies, higher values of this parameter for IO_3_^-^ were noted in the present study than shown by Dai et al. (2004) for carrot grown for 13 weeks in pots located in a greenhouse. This discrepancy may have been caused by the longer time of carrot cultivation in field, different type of soil and the choice of various cultivars.

Generally, the values of I and Se TF in carrot roots (and leaves) can vary to a large extent depending on the cultivation site (climatic zone) and physical and chemical properties of soils (Dai et al., 2004; Uchida et al., 2009; Jolly et al., 2013). An important problem with comparing values of I and Se TF is the diversity of analytical procedures for I and Se determination in soil (substrates in soilless cultures) applied by other authors (Dai et al., 2004; Blasco et al., 2008; Uchida et al., 2009; Jolly et al., 2013). Depending on the procedure (extraction, incubation, or digestion), I or Se forms of various solubility and availability to plants are determined (Yamada et al., 1996, 1999; McNally, 2011; Shetaya, 2011). The methodology of plant analysis is also of some importance, which in the end leads to obtaining different values of I and Se TF. The results of our research indicate that for both KI and KIO_3_, I TF values for leaves were lower than for roots. In the case of Se, this relation was noted only for Na_2_SeO_4_, whereas for Na_2_SeO_3_ the respective values of TF remained at a similar level.

The dose of Se used for fertilization (1 kg Se⋅ha^-1^) was five times lower than of I (5 kg I⋅ha^-1^). Still, irrespective of the research plot, the values of Se TF values for leaves and storage roots were higher than for I. This indicates considerably higher Se mobility, and therefore availability, to plants in the soil environment, since I undergoes very strong sorption in soil (Yoshida et al., 1992; Schlegel et al., 2006; Pless et al., 2007). The described relation is further confirmed by the values of Se and I uptake by leaves and roots. With a five times higher I than Se dose, the largest noted level of I uptake (leaves + roots) was 450 g I⋅ha^-1^ (for KI applied in 2013). In the case of Se, the total Se uptake value (leaves + roots) reached values from 400 g Se⋅ha^-1^ in 2012 to 700 g Se⋅ha^-1^ in 2014 (both for Na_2_SeO_4_). Therefore, the uptake of Se by carrot plants was much more effective than of I.

The transport of SeO_3_^2-^ ions in plants occurs using the same protein transporters as of phosphate ions, while for SeO_4_^2-^, sulfate carriers are engaged (Winkel et al., 2015). Our studies revealed that in combinations fertilized with Na_2_SeO_3_ (alone or together with KI/KIO_3_) the correlation coefficient values for Se and P content in leaves and roots were -0.37* and 0.58*, respectively. This may suggest the inhibition of phosphate transport from roots to leaves under the influence of SeO_3_^2-^. On the other hand, after the application of Na_2_SeO_4_ (alone or with KI/KIO_3_), Se and S content was negatively correlated, with correlation coefficient values of -0.53* and -0.86*, respectively, for leaves and roots. The negative impact of Na_2_SeO_4_ on sulfur content in plants did not, however, affect the biomass productivity, possibly due to the low requirements of this species toward S. The results of S and P determination as well as of other macro- and micro-nutrients in carrot plants will be the subject of other publications.

#### The Effect of Soil and Climatic Conditions

From our study we may assume that reduced I uptake in 2012 was probably a consequence of lower precipitation volume than in the years 2013–2014. In this context, however, the results of Se determination in soil after carrot cultivation are surprising. Its highest content was noted in 2014, while the lowest was in 2012, which was accompanied by the smallest Se uptake by carrot plants. These results indicate that in that latter year Se underwent the highest soil sorption, which reduced its uptake by plants.

Even more surprising were the extremely different concentrations of I and Se in 2012 and 2014, as in these years carrot was cultivated on various parts of the same field. Most probably, diversity with respect to pH, EC and the content of I, Se and organic matter along with diverse weather conditions in 2012–2014 affected these changes in I and Se uptake by plants. Another affecting factor could include speciation processes of I and Se that occur in soil, sometimes leading to its volatilization. We did not, however, study this problem. Additionally, overall soil diversity as well a varying history of cultivation of two separate parts of the field in 2012 and 2014 may have contributed to obtained differences in I and Se content in soil.

There is also no certainty which form of I and Se is analyzed in soil after sample incubation in TMAH. According to Yamada et al. (1996), this method allows determination of the total content of I in soil if the incubation is conducted for 3 h at 90°C. McNally (2011) stated that a similar method of sample incubation with TMAH also allows analysis of the total concentration of I in soil, but not of Se. Ponce de León et al. (2003) demonstrated high applicability of TMAH extraction for Se analysis in soil. Yamada et al. (1999) noted that sample incubation with TMAH conducted for 4 h at room temperature extracts I bound with humic acids. According to Shetaya (2011) sample extraction with TMAH allows analysis of partial or total content of I in soils. Additionally, this author noted that the conditions of incubation including changes of TMAH concentration, extraction time, temperature, or soil particle size did not affect the results of I determination. In our opinion the applied procedure of sample incubation with TMAH provides information of the total absorbed content of I and Se in soil rather than their direct availability to plants in mineral form.

Results of our study indicate that climatic conditions may have a substantial influence on obtaining diverse plant reaction to simultaneous application of I and Se. It cannot be clearly stated which of the monitored weather parameters (with its values varying in the years 2012–2014) contributed mostly to observed changes. Such effect can be determined only in studied conducted in strictly controlled conditions, e.g., in growth chambers

### Iodine and Selenium Interaction with Respect to Their Uptake from Soil

Both tested elements are expelled via leaves through methylation (Rhew et al., 2003; Winkel et al., 2015). We assume that due to the higher values of soil-to-plant TF of Se than I, metabolic pathways responsible for Se methylation were more intensively activated. An increase of I methylation after the application of KI or KIO_3_ alone was also probable. This translated into a diverse effect of fertilization with I or Se alone on the accumulation of Se and I, respectively, in carrot leaves and storage roots. Additionally, the rate of I and Se accumulation in plants was different in each year of the research. In the research by Smoleń et al. (2014) with hydroponic cultivation of lettuce, the introduction of IO_3_^-^ or SeO_4_^2-^ into the nutrient medium had no impact on root uptake and further plant distribution of SeO_4_^2-^ and IO_3_^-^, respectively. Similar results were obtained in a field cultivation of lettuce (Smoleń et al., 2016b). Also, foliar spraying with IO_3_^-^ and SeO_4_^2-^ did not affect root uptake of Se and I from the nutrient medium in carrot cultivation (Smoleń et al., 2014).

### Selenomethionine

After uptake, SeO_3_^2-^ ions in roots are converted into organoselenium compounds (mainly Se-amino acids) and transported in that form to leaves. SeO_4_^2-^ ions, however, after root uptake, are firstly transported through the xylem to stems and then to leaves, in which they are reduced to SeO_3_^2-^. The reduction process is followed by the synthesis of Se-amino acids, e.g., selenocysteine (SeCys) and SeMet. Biosynthesis of organoselenium compounds from SeO_4_^2-^ ions (after previous SeO_3_^2-^ reduction) does not occur in roots, or has a marginal effect, in comparison to Se-amino acid synthesis directly from SeO_3_^2-^ (Zhu et al., 2009; Longchamp et al., 2015; Winkel et al., 2015).

Kápolna et al. (2009), with the use of HPLC–ICP–MS, observed that, regardless of the selenium form (SeO_3_^2-^ or SeO_4_^2-^) administered to plants, the dominant organoselenium compounds in the roots of carrot grown in pots were SeMet and γ-glutamyl-selenomethyl-selenocysteine, while the only one detected in carrot leaves was SeMet. They also demonstrated the presence of free SeO_3_^2-^ or SeO_4_^2-^ ions in carrot leaves and roots, respectively, for the form used in foliar feeding. Their concentration in leaves and roots (expressed as μg Se⋅g^-1^ d.m.) was from several to about a dozen times lower than that of SeMet, and higher (but only in roots) than of γ-glutamyl-selenomethyl-selenocysteine. In our research, we managed to determine only the content of SeMet in carrot leaves and storage roots. With our CE analyzer with DAD detection, we did not reveal the presence of any free SeO_3_^2-^ or SeO_4_^2-^ ions in carrot leaf and storage roots of plants from any combinations of the study (a detailed description of the methodology has been omitted in Section “Plant Analysis”). It can thus be stated that in carrot leaves and storage roots, Se was present in an organic form, with the dominant one being SeMet. An exception was the relatively lower, than in other plots, percentage of Se from SeMet in total Se in leaves and roots of plants fertilized with Na_2_SeO_4_ (alone and combined with KI and KIO_3_), as noted in 2013 and 2014. With no detection of free SeO_4_^2-^ ions, these results indicate that for more favorable weather conditions (occurring in the years 2013–2014 in relation to 2012), more SeO_4_^2-^ ions were transformed into organoselenium compounds other than SeMet, e.g., γ-glutamyl-selenomethyl-selenocysteine or products of the conversion of SeMet or SeCys into volatile selenocompounds: dimethyl selenide and dimethyl diselenide (Zhu et al., 2009; Winkel et al., 2015).

### Biofortification Target – Meeting Consumer Demand for Iodine and Selenium

A basic parameter crucial for assessing the efficiency of simultaneous biofortification of plants with I and Se is RDA, which for these elements is 150 μg I and 55 μg Se for adults, and 200–300 μg I and 60–70 μg Se for pregnant and lactating women, respectively (Food and Nutrition Board – Institute of Medicine, 2000; Andersson et al., 2007). Taking these values into account, the optimum mass ratio of I:Se in food is therefore within 2.7–5.5:1. However, considering the molar mass of both elements, the optimum ratio of I:Se stays within 4.4–8.8:1. Molar ratio seems to be a more reliable indicator applicable in assessing the efficiency of biofortification regarding consumer demand for these nutrients.

In the 3-year research period, the molar ratio of I:Se in a single portion of 100 g f.w. of carrot from the respective research plots was within the following values: (1) Control (2.2–3.9:1), (2) KI (29.4–59.3:1), (3) KIO_3_ (8.4–42.1:1), (4) Na_2_SeO_4_ (0.06–0.009:1), (5) Na_2_SeO_3_ (0.32–0.65:1), (6) KI+Na_2_SeO_4_ (0.45–1.4:1), (7) KIO_3_+Na_2_SeO_4_ (0.31–0.61:1), (8) KI+Na_2_SeO_3_ (2.9–4.1:1), (9) KIO_3_+Na_2_SeO_3_ (1.6–4.4:1). According to this data, even in control plants the molar ratio of I:Se differs from the optimal for consumer need. It should be noted that for that combination the obtained RDA-I values ranged from 6.14% in 2014 to 7.6% in 2012, and of RDA-Se from 8.4% in 2012 to 41.2% in 2014. These results indicate a greater capacity of the control carrot to satisfy consumer demand for Se than I – increasing with the natural content of I and Se in soil.

The most promising results in terms of biofortification purposes were obtained for simultaneous fertilization of Na_2_SeO_3_ with KI or KIO_3_. Not only did the values of RDA-I and RDA-Se oscillate, or slightly exceeded 100%, but the molar ratio of I:Se content in carrot was the closest to optimal – as compared to other combinations. Also, in the above-mentioned research by Smoleń et al. (2016b) with field cultivation of lettuce after the application of KI+Na_2_SeO_3_ and KIO_3_+Na_2_SeO_3_, it was noted that a more optimal ratio of I:Se was obtained in plants fertilized simultaneously with Na_2_SeO_3_ and KI or KIO_3_ than after the application of Na_2_SeO_4_ with KI/KIO_3_.

Future research in this area needs to be expanded to establish the possibility of balancing daily human diets of I and Se by biofortified crop plants. Additionally, it is necessary to investigate the assimilability of these elements by consumers. Tonacchera et al. (2013) demonstrated that after the consumption of vegetables biofortified with I (potatoes, cherry tomatoes, carrots, and green salad), urinary I excretion, one of the major indicators of I status of the human population, increased. Kopeć et al. (2015) revealed higher I assimilability and improvement of this element’s metabolism in rats fed with I-enriched lettuce in relation to those fed with control lettuce and a diet containing mineral KI. In the research by Koronowicz et al. (2016), the inhibition of Caco-2 cancer cell proliferation was demonstrated after treatment with I-biofortified lettuce extract, but not KI as well as I-biofortified lettuce-mediated induction of mitochondrial apoptosis and/or cell differentiation. This study showed 1326 differently expressed Caco-2 transcripts after treatment of biofortified and non-fortified lettuce extract.

## Conclusion

A common health problem is I and Se deficiency in the human and animal population. The increase of I and Se content in plants tissues through various strategies of plant biofortification can be included as one of the ways of innovative crop production. Simultaneous plant enrichment with I and Se stays within the scope of functional food production. Both tested elements are not essential for plants. The results of conducted studies widened the knowledge on its interaction in plants, at the same time proposing the implementation of simultaneous plant fertilization with I and Se into the agricultural practice, mostly in the areas with endemic deficiency of both beneficial elements.

Soil fertilization with KI, rather than KIO_3_, contributed to greater uptake and accumulation of I. For Se, it was the introduction of Na_2_SeO_4_ into the soil that improved Se uptake and increased its content, also in the organic form of SeMet, in carrot leaves and roots. Fertilization with Na_2_SeO_3_, however, stimulated the processes of Se conversion into an organic form, which was reflected by the increased percentage of total Se present in SeMet.

An interaction between I and Se with respect to the rate of accumulation of both beneficial elements was revealed. The clearest relation concerned the negative effect of Se (mainly Na_2_SeO_3_) on I content and uptake. The reverse interaction describing the limiting influence of I on Se uptake and accumulation was most distinctive when KI and Na_2_SeO_3_ were applied together. Interaction of Na_2_SeO_4_ with I with respect to Se uptake, TF values and the content of SeMet was far more diverse within the years of the study.

Our research has demonstrated the possibility of conducting simultaneous soil fertilization with both beneficial elements (I and Se) without the risk of reducing the carrot crop yield. The applied doses of I and Se increased I and Se enrichment of carrot to a level exceeding the possibility of balancing the diet with respect to RDA-I and RDA-Se. In further studies conducted on soils with low content of I and Se as well as in agricultural production, lower doses of both elements should be applied in order to avoid the excessive intake of I and Se with biofortified carrots by both humans and animals.

Combined soil fertilization with I+Se needs to be applied in areas with insufficient concentration of both beneficial elements in soils, and thus in plants/food. Because of higher TF values for Se than I, Se doses for fertilization should be significantly lower than those of I. Despite the lower uptake of Se from Na_2_SeO_3_, application of this compound (and not Na_2_SeO_4_) gave better results in terms of the percentage ratio of Se from SeMet in total Se content in carrot plants.

When cultivating plants on soils intrinsically rich in I and/or Se, additional introduction of I or Se can be conducted but in much lower doses, in order to ensure proper balancing of both beneficial elements content in plants and food. Particular attention should be paid to maintaining the optimal molar ratio of I:Se in plants within the values of: 4.4–8.8:1.

## Author Contributions

SS: Leader of the project, author of the method of carrot biofortification with iodine and selenium, coordinator of field experiments and laboratory analyses, conducted the analysis of results and prepared the manuscript; ŁS: conducted field experiments with carrot cultivation; conducted iodine and selenium analysis using ICP-OES; IL-S: co-founder of the project; involved in the preparation of the research project, author of the method of iodine biofortification of carrot, conducted statistical analysis of results; helped prepare the manuscript; RR: involved in conducting field experiment with carrot cultivation; conducted laboratory analyses using capillary electrophoresis; AKop: helped conducting carrot cultivation; prepared soil and plant samples. EP: helped conducting carrot cultivation; prepared soil and plant samples. RB-K: helped conducting carrot cultivation; prepared soil and plant samples. AKor: helped conducting carrot cultivation; prepared soil and plant samples. JK-D: helped conducting carrot cultivation; prepared soil and plant samples.

## Conflict of Interest Statement

The authors declare that the research was conducted in the absence of any commercial or financial relationships that could be construed as a potential conflict of interest.
